# Alternative polyadenylation drives genome-to-phenome information detours in the AMPKα1 and AMPKα2 knockout mice

**DOI:** 10.1038/s41598-018-24683-7

**Published:** 2018-04-24

**Authors:** Shuwen Zhang, Yangzi Zhang, Xiang Zhou, Xing Fu, Jennifer J. Michal, Guoli Ji, Min Du, Jon F. Davis, Zhihua Jiang

**Affiliations:** 1 Department of Animal Sciences and Center for Reproductive Biology, Washington State University, Pullman, WA USA; 2 College of Animal Sciences and Veterinary Medicine, Huazhong Agricultural University, Wuhan, China; 3 School of Animal Sciences, Louisiana State University, Baton Rouge, LA USA; 4 Department of Automation, Xiamen University, Xiamen, China; 5 Department of Integrative Physiology and Neuroscience, Washington State University, Pullman, WA USA

## Abstract

Currently available mouse knockout (KO) lines remain largely uncharacterized for genome-to-phenome (G2P) information flows. Here we test our hypothesis that altered myogenesis seen in AMPKα1- and AMPKα2-KO mice is caused by use of alternative polyadenylation sites (APSs). AMPKα1 and AMPKα2 are two α subunits of adenosine monophosphate-activated protein kinase (AMPK), which serves as a cellular sensor in regulation of many biological events. A total of 56,483 APSs were derived from gastrocnemius muscles. The differentially expressed APSs (DE-APSs) that were down-regulated tended to be distal. The DE-APSs that were related to reduced and increased muscle mass were down-regulated in AMPKα1-KO mice, but up-regulated in AMPKα2-KO mice, respectively. Five genes: *Car3* (carbonic anhydrase 3), *Mylk4* (myosin light chain kinase family, member 4), *Neb* (nebulin), *Obscn* (obscurin) and *Pfkm* (phosphofructokinase, muscle) utilized different APSs with potentially antagonistic effects on muscle function. Overall, gene knockout triggers genome plasticity via use of APSs, completing the G2P processes. However, gene-based analysis failed to reach such a resolution. Therefore, we propose that alternative transcripts are minimal functional units in genomes and the traditional central dogma concept should be now examined under a systems biology approach.

## Introduction

Information flow from the genome to phenome is dependent on genesis of new RNAs that serve as key as intermediates in this process^1^. In particular, RNA structural diversity and functional dynamics produce a very intricate transfer of genetic information. For example, alternative polyadenylation that results in more than one 3′UTR (untranslated region) end per gene is a critical RNA processing mechanism that affects transcriptome diversity and gene expression dynamics^2^. This mechanism expands RNA flexibility, localization, stability and translational efficiency, causing qualitative, quantitative and/or epigenetic effects that make a gene functionally more diverse^3–5^. Notably, our whole transcriptome termini site sequencing (WTTS-seq) technique^6^ is well-developed method designed to profile the 3′-ends of RNAs in order to understand their roles in regulation of complex phenotypes underlying health and diseases.

Recently, we applied our WTTS-seq method to capture alternative polyadenylation sites (APSs) of transcripts derived from hypothalamus of male rats throughout the progression of diet-induced obesity (DIO). Specifically we used WTTS-seq to understand transcriptome changes that consequently alter information flows from genome to the obese phenotype^7^. Results from this effort indicate that DIO stimulated hypothalamic APSs on protein coding genes enriched for cell (neuron) projection morphogenesis, synaptic transmission, dendrite development, synapse organization, regulation of ion transport, learning, neurotransmitter transport, and regulation of vesicle-mediated transport as top ten summary pathways revealed by the Metascape program^8^. Importantly, independent validation indicated that APSs altered transcriptome expression of plasticity related genes in the hypothalamus. These findings suggest that differentially expressed APSs (DE-APSs) executed function in neuron projection development, synapse organization and neurotransmitter activities, which in turn help explain the phenotypic changes in excess feeding behavior and body weight gain^7^.

Over the last several decades, generation of gene knockout (KO) animals has become a routine approach to determine gene function based on altered phenotypes relative to the wild type (WT) condition. In this context, the adenosine monophosphate-activated protein kinase (AMPK) is a gene that regulates diverse biological function in humans and laboratory animals^9^. There are two α subunits of the mammalian 5′ AMPK gene: AMPKα1 and AMPKα2, which are encoded by *Prkaa1* and *Prkaa2* gene, respectively. Mouse KO models clearly demonstrate that AMPKα2, but not AMPKα1, is related to control of glucose homoeostasis in skeletal muscle^10,11^. In addition, Fu and co-workers^12^ observed that fiber numbers and sizes were reduced by ~25% and ~20% (P < 0.05) in the soleus muscle from AMPKα1 KO mice, relative to WT littermates. In direct contrast, both fiber size and muscle mass were significantly increased in AMPKα2 KO mice (P < 0.05), while the number of muscle fibers remained similar to WT animals. Although these results confirmed that AMPKα1 and AMPKα2 have distinct physiological functions, their underlying mechanisms involved in information detours from genome to phenome in KO animals remain largely unknown.

In the present study, we hypothesized that altered myogenesis in AMPKα1 and AMPKα2 KO mice results from use of different types of APSs in comparison to those in WT animals. Total RNA was extracted from the gastrocnemius muscles of both KO and WT mice. WTTS-seq libraries were constructed and sequenced using the Ion Torrent platform. Characterization of APSs in mice revealed that gene biotypes had significant effects on number of APSs used per gene, within-gene mapping locations and adenine composition in genomic regions downstream of polyA sites. Independent of KO model, we discovered that up-regulated APSs were often located in intronic regions or were extended from exons to introns. Pathway and network analyses of DE-APSs explained the contrasting differences in myogenesis between AMPKα1 and AMPKα2 KO mice. In brief, our data clearly demonstrated that alternative transcripts serve as sensitive and powerful biomarkers that can be used to link genes to their functions via detailed biological processes.

## Results

### APS basics in mouse

**Figure 1:**
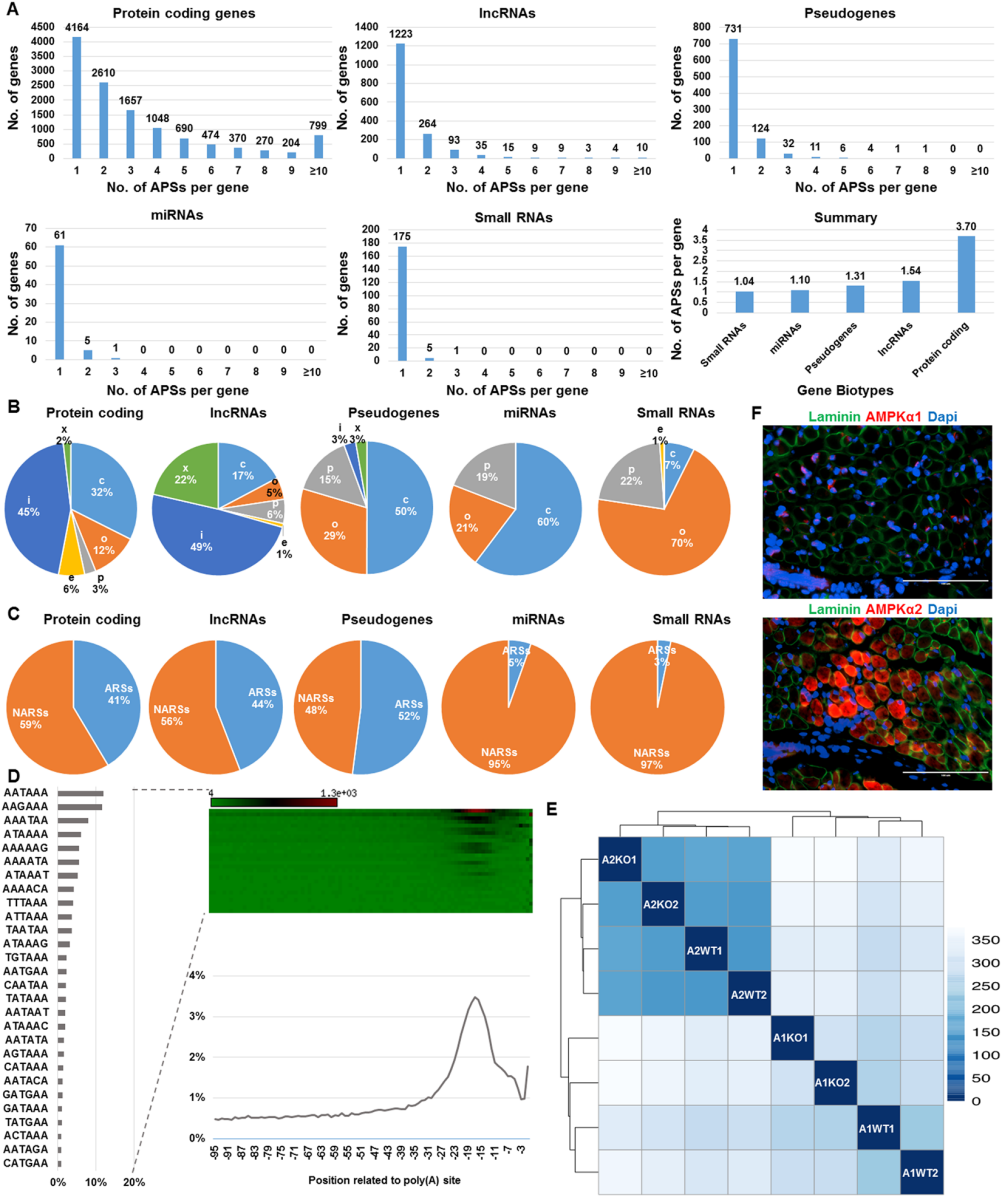
Characterization of APSs in Mice. Effects of gene biotypes on number of APSs per gene (**A)**, within-gene mapping locations with 6 class codes: c, e, i, o, p and x **(B)** and genomic features with ARSs (A rich stretches) and NARSs (non A rich stretches) (**C**). (**D**) PolyA signal distributions in mouse. **(E)** Heat map and dendrogram of sample-to-sample distances among 8 libraries based on APS profiles. Two KO and two WT mice were used for each case in the present study. **(F)** Expression of both AMPKα1 and AMPKα2 in skeletal muscle of WT mice at 10 days of age.

Using 16 reads per site as a cutoff, we identified a total of 56,483 APSs expressed in murine gastrocnemius muscles (Supplementary Table S1). Among them, 49,783 APSs were assigned to the currently annotated genes in the species. Approximately 66% (8,122/12,286) of protein coding genes use more than one polyA site (Fig. 1A). In comparison, ~27% (442/1,665) of lncRNAs (long non-coding RNAs), ~20% (179/910) of pseudogenes, ~9% (6/67) of miRNAs (microRNAs) and 3% (6/181) of small RNAs use more than one polyA site (Fig. 1A). Consequently, the number of APSs per gene were greatest in protein coding genes (3.70), but were 1.54, 1.31, 1.10 and 1.04 for lncRNAs, pseudogenes, miRNAs and small RNAs, respectively (Fig. 1A). Small RNAs include scRNAs (small conditional RNAs), snoRNAs (small nucleolar RNAs), snRNAs (small nuclear RNAs), telomerase RNAs, tRNAs (transfer RNAs) and Y chromosome RNAs.

In addition to APS use frequencies described above, gene biotypes also had significant effects on their within-gene locations (χ^2^ = 6114.1, df = 20, p-value < 2.2e-16 among six class codes, Fig. 1B) and downstream genomic features (χ^2^ = 214.58, df = 4, p-value < 2.2e-16 between ARSs (A-rich stretches) and NARSs (non A-rich stretches) (Fig. 1C). These six class codes were defined by the Cuffcompare tool (version 2.2.1)^13^, which were further classified into two clusters. The non-conventional APSs were located either in introns (i, completed in the intronic regions and e, extended from exonic regions to intronic regions with at least 10 bp) or on antisense strand exons (x, exonic regions, but with opposite direction). The conventional APSs were majorly derived from exons (c, confined in exonic regions; o, exonic regions with extension and p, located within 2 kb downstream of reference transcripts).

As shown in Fig. 1B, these two clusters of APSs were distributed almost equally for protein coding genes, but the ratio shifted to almost 75:25 for lncRNAs. However, pseudogenes, miRNAs and small RNAs rarely used the non-conventional types of APSs (no more than 1%). More than 90% of small RNAs use distal APSs (o and p sites) compared to 40–44% for miRNAs and pseudogenes. Pseudogene APSs were distributed equally between ARSs and NARSs. This ratio was almost 40 ARSs:60 NARSs for protein coding genes and lncRNAs (Fig. 1C) and approximately 4 ARSs:96 NARSs for miRNAs and small RNAs (Fig. 1C).

Similar to other species, AAUAAA is the most dominant polyA signal in the mouse transcriptome (Fig. 1D). PolyA signals were mainly located between −15 and −30 bp upstream of tentative polyadenylation sites. Of 56,483 APSs, 49,369 and 50,017 sites were expressed in AMPKα1 KO – WT pair and AMPKα2 KO – WT pair, respectively (Supplementary Table S1). The sample-to-sample distances clearly separated our experiments into both pairs and into KO and WT groups within each model case (Fig. 1E). Overall, transcriptome similarity was higher between the two WT groups than between the two KO groups. Furthermore, we detected the expression of both *AMPKα1* and *AMPKα2* in skeletal muscle in WT mice at age of 10 days by IHC (Immunohistochemical staining) assay (Fig. 1F).

### Gain and loss of DE-APSs in AMPKα1 KO mice

**Figure 2:**
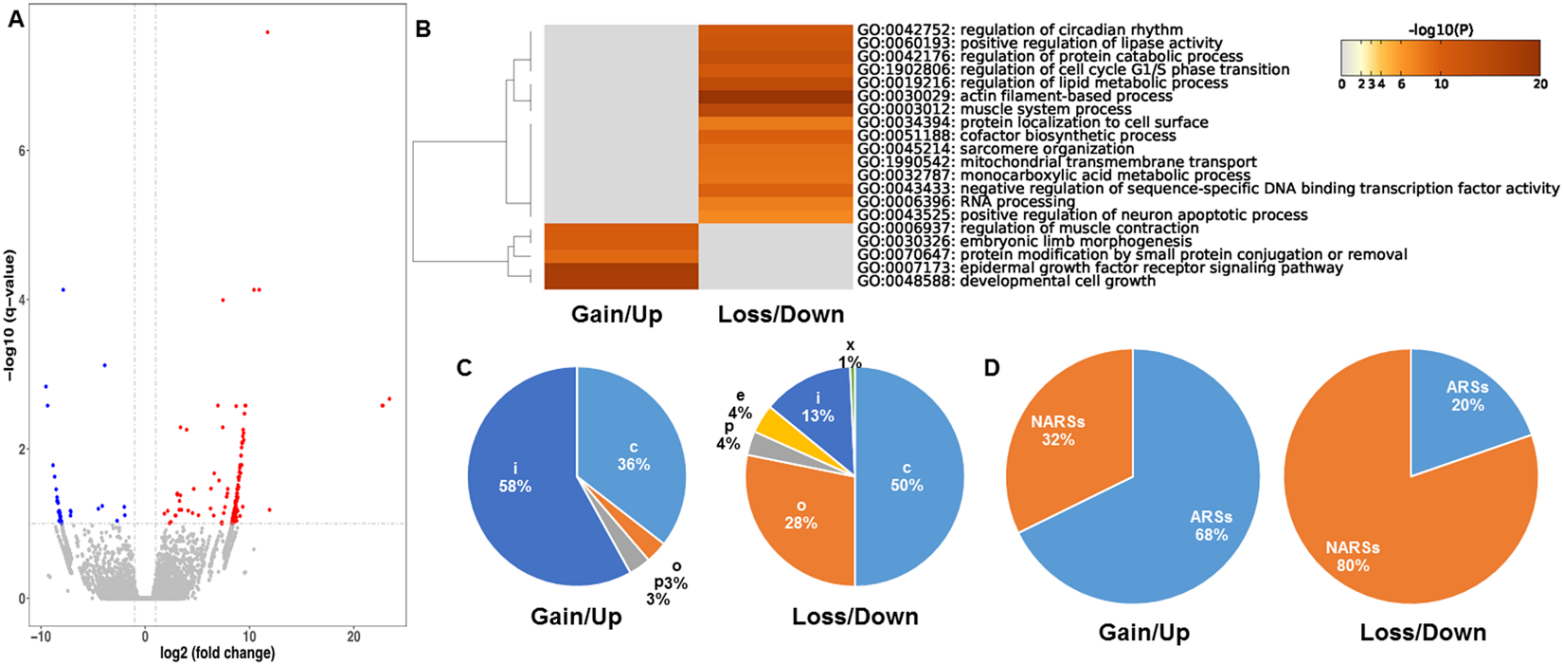
Characterization of DE-APSs between two AMPKα1 KO and two WT mice. (**A**) Volcano plot of APSs separating gained or up-regulated APSs (left) from lost or down-regulated APSs (right) in AMPKα1 KO mice. **(B)** Gene-enriched pathways with gained/up-regulated and lost/down-regulated DE-APSs. **(C)** Class code usages between gained/up-regulated and lost/down-regulated DE-APSs. **(D)** Comparison of downstream genomic regions between gained/up-regulated and lost/down-regulated DE-APSs.

Among 49,369 APSs expressed between two AMPKα1 KO and two WT animals, 205 were DE-APSs (adjusted P < 0.1) (Fig. 2A and Supplementary Table S2). Based on log2 fold changes, AMPKα1 KO mice had 28 DE-APSs that were not expressed in WT mice and 10 DE-APSs that were up-regulated. In addition, 132 APSs were not expressed in AMPKα1 KO mice and 35 DE-APSs were down-regulated in comparison to WT mice. Pathway enrichment analysis was conducted with the DE-APSs associated with protein coding genes, including 31 DE-APSs/31 DE-genes exclusively expressed or up-regulated, and 142 DE-APSs/141 DE-genes lost or down-regulated in AMPKα1 KO mice.

As shown in Fig. 2B, AMPKα1 KO mice had three pathway clusters down-regulated in 1) muscle and lipid processes, such as actin filament-based process, muscle system process, sarcomere organization, lipase activity and regulation of lipid metabolic process; 2) DNA, RNA and cellular processes, such as sequence-specific DNA binding transcription factor activity, RNA processing, circadian rhythm and cell cycle G1/S phase transition; and 3) protein, transport and metabolic processes, such as protein catabolic process, protein localization to cell surface, monocarboxylic acid metabolic process, mitochondrial transmembrane transport, cofactor biosynthetic process, and neuron apoptotic process. On the other hand, AMPKα1 KO mice also had up-regulated pathways related to regulation of muscle contraction, embryonic limb morphogenesis, protein modification by small protein conjugation or removal, epidermal growth factor receptor signaling pathway and developmental cell growth.

For the 31 protein coding DE-APSs that were exclusively expressed or up-regulated in AMPKα1 KO mice, 58% (18/31) originated from intronic sites. In contrast, only 13% (19/142) of DE-APSs that were lost or down-regulated due to AMPKα1 knockout (χ^2^ = 33.101, df = 5, p-value = 3.594e-06) were derived from intronic sites (Fig. 2C). In addition, the exclusively expressed or up-regulated DE-APSs were often associated with ARSs (68%, 21/31), while the lost or down-regulated DE-APSs were mostly adjacent to NARs (80%, 114/142) (χ^2^ = 26.589, df = 1, p-value = 2.517e-07) (Fig. 2D). Furthermore, AMPKα1 KO mice had three pseudogene DE-APSs that were exclusively expressed or up-regulated, but lost nine pseudogene DE-APSs plus three lncRNA DE-APSs compared to WT mice (Supplementary Table S2).

### Up- and down-regulated DE-APSs in AMPKα2 KO mice

**Figure 3:**
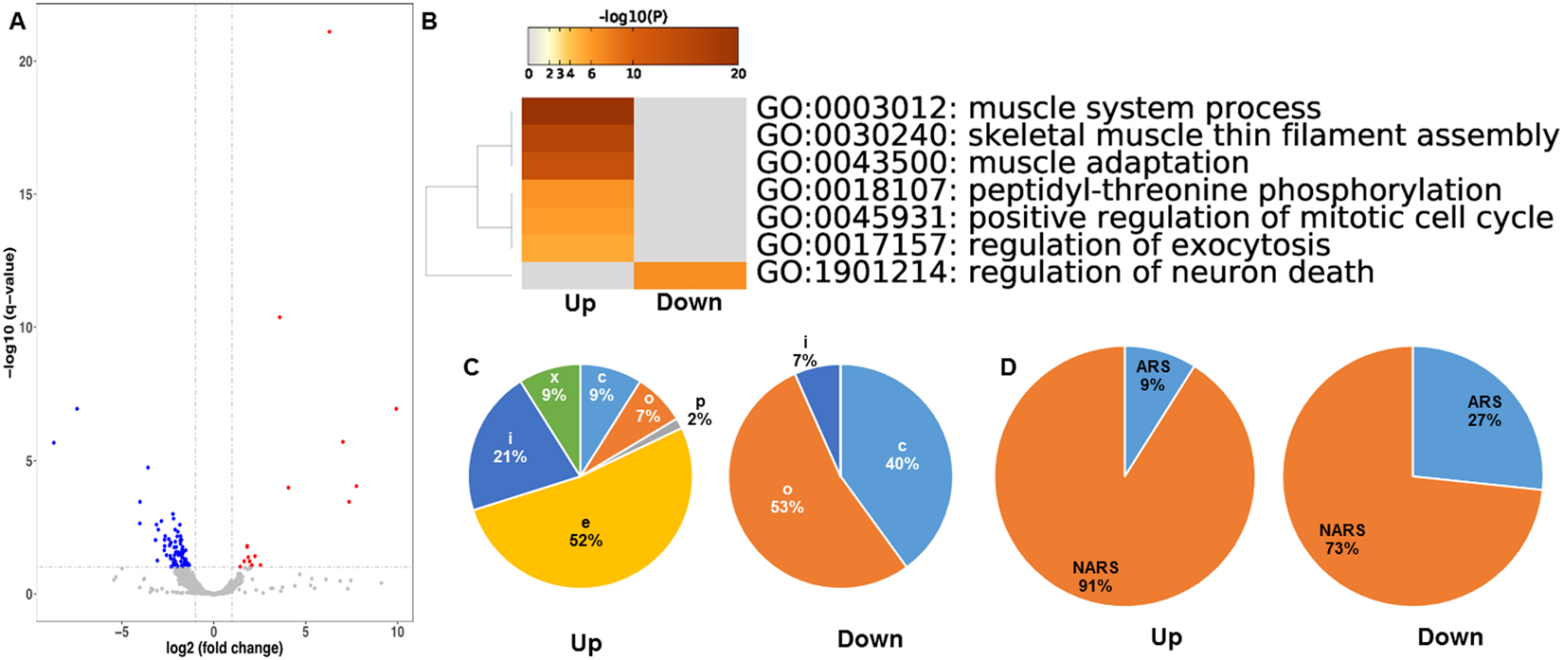
Characterization of DE-APSs between two AMPKα2 KO and two WT mice. (**A**) Volcano plot of APSs separating up-regulated APSs (left) from down-regulated APSs (right) in AMPKα2 KO mice. **(B)** Gene enriched pathways with up- and down-regulated DE-APSs. **(C)** Class code usages between up- and down-regulated DE-APSs. **(D)** Comparison of downstream genomic regions between up- and down-regulated DE-APSs.

Among 50,017 APSs expressed between two AMPKα2 mice and two WT animals, 101 were identified as DE-APSs (Fig. 3A and Supplementary Table S3). Of these, 84 were up-regulated and 17 were down-regulated. Among them, 67 up-regulated DE-APSs and 15 down-regulated DE-APSs were assigned to 44 and 14 DE-genes, respectively. The dramatic difference between up-regulated DE-APSs (67) and up-regulated DE-genes (44) was due to the fact that five genes: *Myh2* (myosin, heavy polypeptide 2, skeletal muscle, adult), *Neb* (nebulin), *Ptp4a2* (protein tyrosine phosphatase 4a2), *Tnnt3* (troponin T3, fast skeletal type) and *Ttn* (titin) had more than one differentially expressed polyA site. In particular, *Ttn* had a total of 17 significant DE-APSs. As shown in Fig. 3B, pathway enrichment analysis revealed that muscle system process, skeletal muscle thin filament assembly and muscle adaptation were significantly up-regulated in AMPKα2 knockout mice. The only down-regulated pathway was regulation of neuron death.

The 67 up-regulated DE-APSs located in protein coding genes mainly extended from exons to introns (e class code with 52%), followed by i class code with 21% (Fig. 3C). In contrast, the 15 down-regulated DE-APSs were dominantly located in distal regions −53% assigned to o class code and 40% designated as c class code. As such, mapping regions between up- and down-DE-APSs differed significantly (χ^2^ = 35.098, df = 5, p-value = 1.439e-06). However, ARSs and NARSs were similarly distributed in both groups of DE-APSs (χ^2^ = 2.1269, df = 1, p-value = 0.1447). In addition, one lncRNA DE-APS, two pseudogene DE-APSs, and one snoRNA DE-APS were up-regulated, and one pseudogene DE-APS was down-regulated in AMPKα2 KO mice compared to WT controls (Supplementary Table S3).

The total RNA samples derived from these two AMPKα2 KO mice and two WT animals were also profiled for 5′-ends of transcripts using our newly developed whole transcriptome start site sequencing (WTSS-seq) method. Among 42,207 ASSs (alternative start sites) identified (Supplementary Table S4), 343 reached the significance level with adjusted P < 0.1 so that they were considered as DE-ASSs, including 96 down- and 247 up-regulated in KO in comparison to WT mice. We observed that DE-genes associated with these DE-ASSs were also enriched for muscle related pathways, such as actin filament-based process, muscle structure development, striated muscle cell differentiation and striated muscle hypertrophy listed as 1st, 2nd, 3rd and 15th pathway among top 20 “summary” pathways in order, respectively (Supplementary Figure S1A). No doubt, AMPKα2 KO mice had more up- (26, 24, 14 and 6) than down-regulated DE-genes (9, 7, 0 and 0) contributed to these four pathways (Supplementary Figure S1B). In addition, several development pathways were also enriched in AMPKα2 KO mice. In brief, our 5′-end profiles further confirmed that AMPKα2 KO triggered whole body growth and muscle functions.

### Functional divergence between AMPKα1 and AMPKα2 KO mice

**Figure 4:**
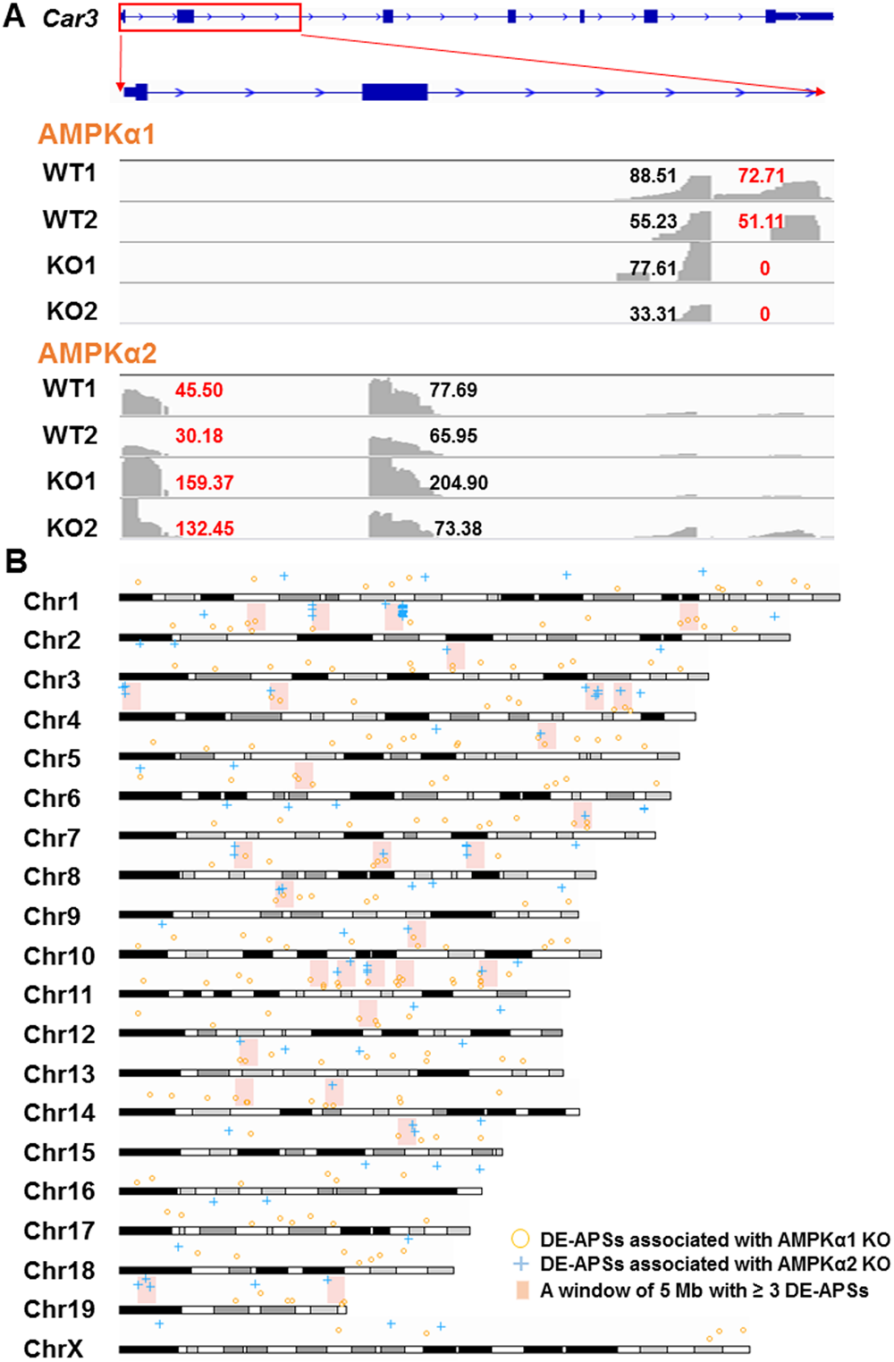
Characterization of DE-APSs between AMPKα1 KO and AMPKα2 KO mice. (**A)** *car3* was used as an example to show that knockouts trigged use of different polyA sites for functional detours. A polyA site in intron 2 was lost in AMPKα1 KO mice while a polyA site in intron 1 (extended from exon 1) was up-regulated in AMPKα2 KO mice in comparison to WT animals. For both knockout cases, each had one site as reference without significant difference. The DESeq normalized count for each site is also presented. **(B)** DE-APS clusters were identified along each murine chromosome, which required at least 3 DE-APSs within a 5-Mb window.

As shown in Supplementary Tables S2 and S3, the DE-APSs revealed between KO and WT were model-specific. However, *Car3* (carbonic anhydrase 3), *Mylk4* (myosin light chain kinase family, member 4), *Neb* (nebulin), *Obscn* (obscurin, cytoskeletal calmodulin and titin-interacting RhoGEF) and *Pfkm* (phosphofructokinase, muscle) were differentially expressed in both models (Supplementary Table S5). The *Neb* gene had 4 DE-APSs, including one up-regulated in AMPKα1 KO and three up-regulated in AMPKα2 KO mice in comparison to wild-type controls. Among the remaining four genes, each had two DE-APSs with one lost in AMPKα1 KO and one up-regulated in AMPKα2 KO. The details are shown in Fig. 4A using *Car3* gene as an example. Overall, these five genes were frequent users of APSs totaling 354, ranging from 12 for *Car3* to 187 for *Neb* (Supplementary Table S5). We observed that distal APSs associated with these five genes were all expressed. In addition, these 12 DE-APSs for both models were classified into c (3), e (6) and i (3) according to their mapping locations. *In silico* validation confirmed all c and e types of DE-APSs. For three intronic DE-APSs, two were strongly supported by ESTs (expressed sequence tags) in mouse (Supplementary Table S5).

It seems that there were different genome regions targeted by AMPKα1 and AMPKα2 knockouts (Fig. 4B). Among 303 DE-APSs for both cases with map locations, over one third (115) were relatively clustered in 29 regions set up with a window of 5 Mb with at least 3 DE-APSs. In particular, chromosomes 2, 4, 8 and 11 were enriched with 4, 4, 3 and 5 such regions, respectively (Fig. 4B). The specific regions targeted by AMPKα1 knockout included two on chromosome 2, one on chromosome 6, two on chromosome 11, one on chromosome 12 and one on chromosome 14. A total of five regions were enriched for AMPKα2 knockout with one on chromosome 2, two on chromosome 4, one chromosome 8 and one on chromosome 19 (Fig. 4B).

**Figure 5:**
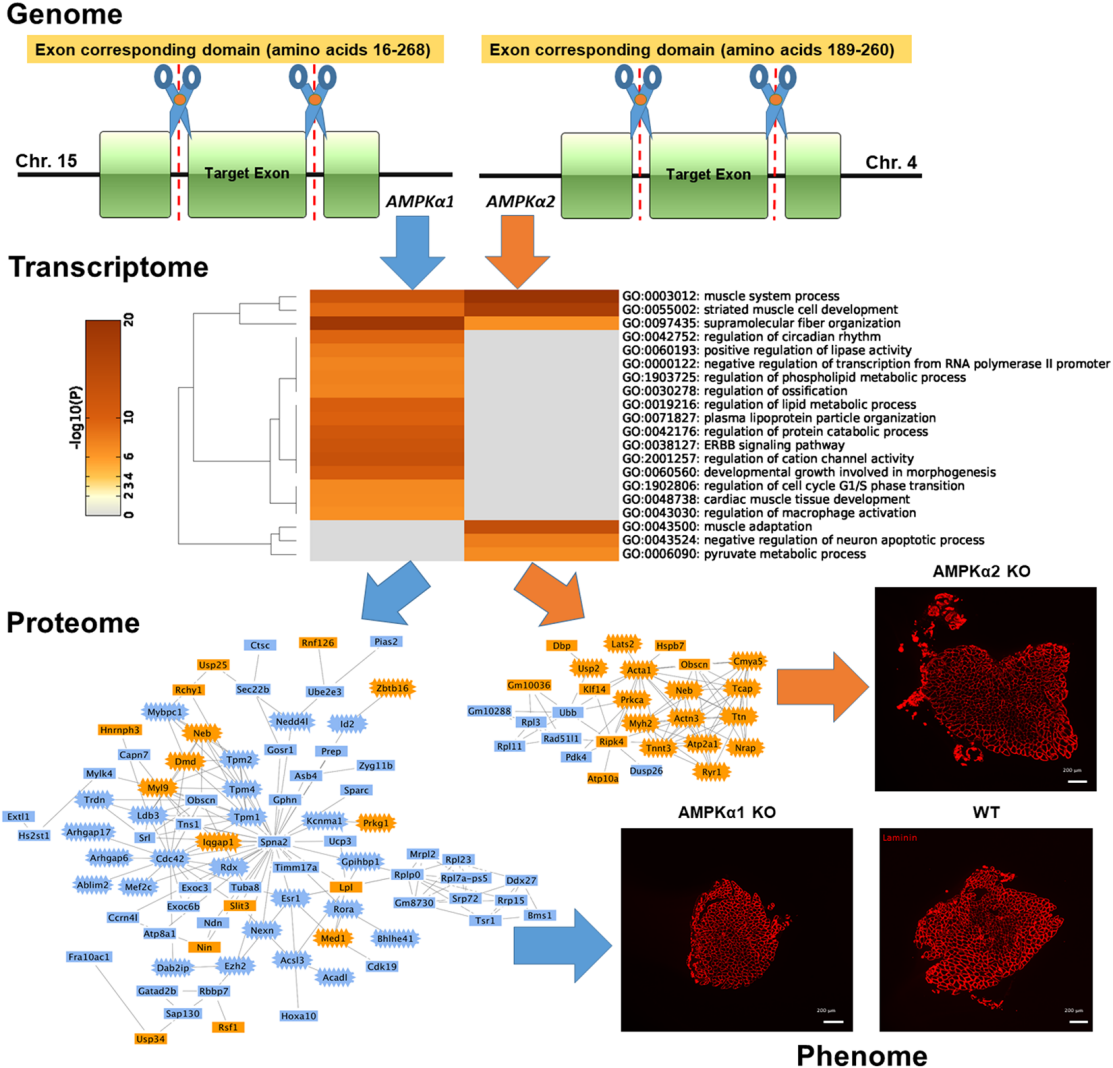
Information flow from genome to phenome in knockout models. The genomic DNA regions coding for partial catalytic domains of AMPKα1 and AMPKα2 proteins were abolished, which caused dynamics of information flows in both models via APS-associated genes enriched for muscle systems. Lost and down-regulated DE-APSs (in blue color) were dominant in AMPKα1 KO mice, while up-regulated DE-APSs (in yellow color) were influential in AMPKα2 KO mice. The information flows matched well with opposite phenotypic changes between both models. Proteins crowned in the networks are associated with muscle system.

Collection of the top 20 enriched pathways based on the full sets of DE-genes associated with both up- and down regulated DE-APSs further revealed the functional divergence between AMPKα1 KO and AMPKα2 KO mice (Fig. 5). The KO models only shared three common pathways: muscle system process, striated muscle cell development and supramolecular fiber organization. Among the remaining pathways, 14 are specific to AMPKα1 KO and 3 to AMPKα2 KO (Fig. 5). Parallel to the pathway analysis, protein-protein interaction (PPI) networks were also mainly enriched from down-regulated genes (over 80%) in AMPKα1 KO, but up-regulated genes (75%) in AMPKα2 KO (Fig. 5). Notably, AMPKα1 KO mice had 24 down- and 7 up-regulated proteins specific to muscle and lipogenesis functions. For AMPKα2 KO mice, 14 out of 21 up-regulated proteins were relevant to muscle processes.

## Discussion

Approximately ten years ago, the International Knockout Mouse Consortium’s goal was to inactivate all the genes harbored in the mouse genome using both gene trapping and gene targeting approaches to facilitate functional studies^14^. Approximately 95% of these tasks have been accomplished^15^. Unfortunately, most of these KO models remain uncharacterized. In the present study, we investigated AMPKα1 KO and AMPKα2 KO mice. For the former model, a region of DNA sequences that code for amino acids 16–268, a part of the catalytic domain of *AMPKα1* gene, were deleted^16^. For the latter model, Viollet and colleagues^11^ abolished the AMPKα2 catalytic domain covering amino acids 189–260 (Fig. 5). We collected gastrocnemius muscle samples from both KO and WT mice and profiled APSs. Our results revealed that AMPKα1 KO mice lost more APSs than gained, while AMPKα1 KO mice had more up-regulated than down-regulated APSs (Figs 2 and 3). Further analysis showed that down-regulated APSs in the former model and up-regulated APSs in the latter model were derived from unique sets of genes enriched for muscle-related processes (Fig. 5), which, in turn, can explain why muscle area was reduced in AMPKα1 KO mice, but increased in AMPKα2 KO mice (Fig. 5). In brief, profiling APSs is a sensitive and powerful approach that can be used to detail the information flow from genome to transcriptome/proteome to phenome and explain altered phenotypes between KO and WT animals.

Mapping reads to the genome also allowed us to collect number of reads per gene and thus pursue gene-based analysis (Supplementary Table S6). Among 15,378 genes collected with at least 16 reads per gene, a total of 123 and 82 were as DE-genes (adjusted P < 0.1) in AMPKα1 KO and AMPKα2 KO mice, respectively as compared to WT animals. When only protein coding genes were considered, the numbers of up- and down-regulated DE-genes were 25 and 71 in AMPKα1 KO mice, and 47 and 22 in AMPKα2 KO mice, respectively (Supplementary Figure S2). On the other hand, APS-based analysis revealed 31 up- and 141 down-regulated DE protein coding genes in the former model and 44 up- and 14 down in the latter model, respectively (Supplementary Figure S2). These results indicated that both APS- and gene-based analyses do not reveal the same sets of DE-genes. As shown in Supplementary Figure S2, the common sets of DE-genes were variable, ranging 14–72%. As such, the DE-genes revealed by the gene-based analysis were enriched for pathways that were quite different from those revealed by the APS-based analysis (Figs 2 and 3 and Supplementary Figure S2). In particular, the gene-based approach did not detect pathways that can explain the phenotypic changes in muscle development between the two KO models (Fig. 5). Overall, only one enriched pathway in AMPKα2 KO mice was relevant to muscle, i.e., cardiac muscle hypertrophy. Therefore, gene-based analysis had much less power than APS-based analysis in discovery of functionally relevant pathways that can explain the phenotypic changes.

Another striking beauty of APS-based analysis observed in the present study is that it discovered five DE-genes shared by both KO models, including *Car3*, *Mylk4*, *Neb*, *Obscn* and *Pfkm*. These DE-APSs (12 in total) were all model-specific (Supplementary Tables S2 and S3). However, gene-based analysis only revealed that *Mylk4* and *Obscn* genes were up-regulated in AMPKα2 KO mice with adjusted P = 0.002 and adjusted P = 0.043, respectively (Supplementary Table S6). Most importantly, these five genes play significant roles in muscle system processes. For example, carbonic anhydrase 3 (Car3) serves as a mesodermal marker for early muscle development^17^. Particularly, this enzyme is abundantly expressed in the type I (slow twitch) skeletal muscle^18^. Myosin light chain kinase 4 (Mylk4) is a member of the myosin light-chain kinase family of serine/threonine-specific protein kinases that phosphorylate the regulatory light chain of myosin II^19^, but its function remains unknown. Both nebulin (Neb) and obscurin (Obscn) are members of the giant protein family and play important roles in contractility of skeletal muscle^20,21^. Phosphofructokinase muscle (Pfkm) is an enzyme that is involved in breakdown of glycogen. As such, Pfkm deficiency often causes glycogen storage disease of skeletal muscle^22^. For *Car3*, *Mylk4*, *Obscn* and *Pfkm* genes, our results clearly support “down-expression – reduced phenotype” and “up-expression – increased phenotype” as the DE-APS action mode. However, the specific DE-APS action mode in the *Neb* gene were “up-expression – reduced phenotype” in AMPKα1 KO mice, but “up-expression – increased phenotype” in AMPKα2 KO mice. How DE-APSs within the same gene have different action modes needs further investigation.

For protein coding genes, conventional (with class codes of c, o and p) and non-conventional (with class codes of e, i and x) accounted for 47% and 53% of the APSs in the collective dataset, respectively (Fig. 1B). However, the distribution of conventional and non-conventional APSs changed to 42% and 58% for up- and 82% and 18% for down-regulated DE-APSs in AMPKα1 KO mice (Fig. 2C), and 18% and 82% for up- and 93% and 7% for down-regulated DE-APSs in AMPKα2 KO mice, respectively (Fig. 3C). The same trends were confirmed based on the results annotated with the HOMER program (Supplementary Table S7). For example, distal (within 3′UTR and TTS) and proximal APSs (non-distal APSs) accounted for 47% and 53%, respectively, based on the whole dataset, but changed to 32% and 68% for up- and 75% and 25% for down-regulated DE-APSs in AMPKα1 KO mice (Fig. 2C), and 6% and 94% for up- and 93% and 7% for down-regulated DE-APSs in AMPKα2 KO mice, respectively (Supplementary Table S7). These results indicate that up-regulated DE-APSs tend to use more non-conventional or non-distal sites while down-regulated DE-APSs were dominantly derived from conventional or distal sites. Therefore, gene knockout may affect different regulatory mechanisms that drive the phenotypic detours.

In addition to alternative polyadenylation, alternative promoter, alternative splicing and RNA editing also significantly contribute to transcriptome diversity and dynamics^23–25^. In the present study, two AMPKα2 KO and two WT animals were also profiled for both 5′- and 3′-ends of transcripts using either WTSS-seq or WTTS-seq method. As shown in Supplementary Tables S3 and S4, both profiles generated very consistent results to support the claim that AMPKα2 KO triggered usage of more alternative transcription start sites and alternative polyadenylation sites than WT animals. The numbers of up- and down-regulated DE-APS were 247 and 96 revealed by WTSS-seq and 84 and 17 detected by WTTS-seq method, respectively. As such, more top “summary” pathways were enriched for the former profiles than the latter profiles (Supplementary Figure S1 and Fig. 3). However, both datasets discovered the same story that the up-regulated DE-genes are responsible for increased muscle function in AMPKα2 KO. Nevertheless, these results also provided hints that gene knockout mice may use a variety of mechanisms to detour the genetic information transfer from genome to phenome.

In conclusion, our present study confirmed that AMPKα1 and AMPKα2 execute different physiological functions although each serves as the α unit of the kinase enzyme. More APSs were lost than gained in AMPKα1 KO mice, which resulted in reduced muscle area. In contrast, knockout of AMPKα2 caused more APSs to be up-regulated, increasing muscle area compared to WT mice. First, these results demonstrated that deletion or deficiency of genes can produce either gain or loss of function, depending on definition and types of phenotypes. Second, alternative transcripts within a gene may serve as different functional units. In particular, different transcripts may be involved in opposite processes that result in either increase or decrease of phenotypes. Lastly, the central dogma of molecular biology in which DNA makes RNA and makes protein^26^ may be oversimplified. Our results indicate that the intricate coordination of alternative transcripts affect phenotypes. Nevertheless, profiling of alternative transcripts is a sensitive and powerful approach to link genome to phenome for radically understanding genetic complexity of complex phenotypes.

## Methods

### Animals and tissue sampling

Two AMPKα1^−/−^ 129S2/SvPas (AMPKα1 KO) and two AMPKα2^−/−^ C57BL/6 (AMPKα2 KO) mice and their WT controls (each with two animals) used in the present study were generated as described previously^11,16^. All animals were purchased from the Jackson Laboratory as described by Fu and colleagues^12^ and handled in accordance with protocols approved by the Animal Care and Use Committee of Washington State University. AMPKα1 KO mice ~6 weeks of age and AMPKα2 KO mice ~8 weeks of age were euthanized by carbon dioxide asphyxiation followed by cervical dislocation. The gastrocnemius muscles were immediately removed, flash-frozen in liquid nitrogen, and stored at −80 °C. RNA was isolated from the muscles following standard procedures. Briefly, muscle was removed from −80 °C and placed in a mortar chilled with liquid nitrogen. The tissue was ground to a fine powder with a pestle, transferred to a Potter-Elvehjem tissue grinder, homogenized with TRIzol Reagent (Life Technologies Corp., Carlsbad, CA) and total RNA isolated according to the manufacturer’s instructions. Contaminating DNA was removed from total RNA with DNase (TURBO DNA-free kit, Life Technologies Corp., Carlsbad, CA). Total RNA quantity was measured with the Quant-iT RiboGreen RNA Assay Kit (Life Technologies Corp., Carlsbad, CA) and quality assessed by fragment analysis (Advanced Analytical Technologies, Inc., Ankeny, IA).

### Immunohistochemical staining (IHC) assays

Soleus muscle samples were collected from 3 month old WT, AMPKα1 KO, and AMPKα2 KO mice. Ttransversus abdominis muscle samples were collected from 10-day old WT mice. Muscle samples were fixed in 4% paraformaldehyde for 4 h at 4 °C, incubated in PBS with 30% sucrose overnight, embedded in OCT (optimal cutting temperature) compound, and then frozen in liquid nitrogen-cooled isopentane. Sections (7 µm thick) were incubated in blocking buffer containing TBS (Tris-buffered saline), 5% goat serum, and 0.2% Triton X-100 for 2 h. Antigen retrieval for IHC employing anti-AMPKα1 antibody (ABIN737886 Antibodies-Online Inc., 1:200) or anti-AMPKα2 antibody (ABIN2855639 Antibodies-Online Inc., 1:200) was performed by heating sections in citrate buffer (pH 6.0) for 20 min before blocking. Blocked sections were incubated overnight at 4 °C in primary antibodies diluted in blocking buffer, rinsed 3 times in TBS containing 0.2% Triton X-100, and then incubated in appropriate fluorophore-conjugated secondary antibodies diluted in blocking buffer for 1 h at room temperature. Sections were then rinsed 3 times in TBS containing 0.2% Triton X-100 and mounted in mounting medium containing DAPI (4′,6-diamidino-2-phenylindole). Images were captured using an EVOS microscope. Additional primary antibody and dilution used in IHC includes rat anti-laminin antibody (4H8-2 Enzo, 1:200). Secondary antibodies were used at 1:500 dilution in IHC, which include goat anti-rabbit Alexa Fluor 555 (#4413) and goat anti-rat Alexa Fluor 488 (#4416) purchased from Cell Signaling.

### Preparation of WTTS-seq libraries and sequencing

In the present study, two male KO and two male WT mice were used per gene model. For each individual, 2.5 μg of total RNA derived from muscle were chemically fragmented with RNA fragmentation buffer (AM8740, Ambion). After that, poly(A)+ RNA was enriched by Dynabeads oligo(T) magnetic beads (61002, Ambion). Reverse transcription with SuperScript III Reverse Transcriptase (18080, Invitrogen) was used to synthesize first cDNA strand with integration of both 5′adaptor and 3′adaptor. After all RNA molecules were removed by both RNases H (M0297L, NEB) and I (EN0601, Thermo Scientific), solid-phase reversible immobilization beads were used to select 300–600 bp first-strand cDNA molecules (A63880, Beckman Coulter), and second-strand cDNA was synthesized by PCR. Size selection was repeated as described above and the final libraries were sequenced using an Ion PGM™ system at Washington State University.

### Read quality control, mapping and processing

Sequencing of 8 WTTS-seq libraries yielded a total of 43,846,314 raw reads, ranging from 2,988,420 to 8,248,846 reads per sample. Quality control was conducted using FASTX Toolkit version 0.0.13.1 to remove reads that had more than 50% of all bases with quality scores less than 10, leaving 43,728,315 reads (99.73%) for further analysis. As our WTTS-seq method is strand-specific, all Ts (thymine(s)) at beginning of each read were trimmed using our own Perl script. We retained trimmed reads with at least 16 bp for mapping, resulting in a total of 41,981,802 qualified reads. The mouse reference genome in FASTA format and annotation file in GFF format were downloaded from NCBI (https://www.ncbi.nlm.nih.gov/genome/52). Read mapping was completed using TMAP (version 3.4.1, https://github.com/iontorrent/TMAP). The first nucleotide of each trimmed read tentatively served as the polyadenylation adjacent site, but when it was clustered with others within a window of 24 nucleotides, it was designated as an APS site^27,28^.

### Characterization of APSs in mice

The Cuffcompare tool (version 2.2.1)^13^ was used to assign genome, gene and within-gene mapping information to each clustered APS site (Supplementary Table S1). Genome information included scaffold number, strand direction and 5′- and 3′-end coordinates on each chromosome. Gene information had ID number, strand, biotype, symbol and transcript ID for each APS. In terms of gene biotypes, we targeted APSs assigned to protein coding genes, long noncoding RNAs (lncRNAs), microRNAs (miRNAs), pseudogenes and small RNA genes. For within-gene information, we focused on APSs assigned to class code c (confined in exonic regions), e (extended from exonic regions to intronic regions with at least 10 bp), i (completed in the intronic regions), o (exonic regions with extension), p (located within 2 kb downstream of reference transcripts) and xAPSs (exonic regions, but with opposite direction). In addition, we also characterized genomic regions 30 bp downstream of APSs based on their adenine contents as ARS-APSs (A-rich stretches-APSs) and NARS-APSs (non-A rich stretches-APSs). An ARS region contained at least 7 consecutive As or 8 As within a 10 bp window. As well, 28 polyA signals predicted in other species^29–33^ were used to determine their potential appearance in genomic region of 100 bp upstream of each APS.

### Preparation of WTSS-seq libraries and sequencing

WTSS-seq library started with 1 µg of total RNAs for depletion of rRNA using RiboCop Rrna depletion kit (Lexogen), followed by reverse transcription with SuperScript III Reverse Transcriptase (18080, Invitrogen) to synthesize first cDNA strand with integration of both 5′adaptor and 3′adaptor in the reaction. In the process, the rRNA depleted RNA was mixed with 1 µl of 100 µM 5′-adaptor (switching primer) and 1 µl of 100 µM 3′-adaptor (semirandom primer). The mixture was heated at 65 °C for 5 min and chilled on ice for 2 min and repeated. After that, 4 µl of of 5x First-Strand Buffer, 2.5 µl Dntp(10 mM), 1 µl SuperScript III Reverse Transcriptase (200 units/µl), 1 µl of RNase OUT(100 units/µl) and 1 µl DTT(0.1 M) were added. Reverse transcription was set as 40 °C for 90 min and 70 °C for 15 min. After all RNA molecules were removed by both RNases H (M0297L, NEB) and I (EN0601, Thermo Scientific), 250–500 bp of the first strand cDNA molecules were selected by solid-phase reversible immobilization beads (A63880, Beckman Coulter) and then used for PCR to synthesize second-strand cDNA. PCR conditions were initial denaturation at 98 °C for 30 sec; 20 cycles of 98 °C for 10 sec, 50 °C for 30 sec, and 72 °C for 8 sec; and final extension at 72° for 10 min. The libraries were sequenced using an Ion PGM™ Sequencer at Washington State University after final size selection was performed with same range descripted above.

### Data analysis and availability

The DESEq. 2 package in R^34^ was used to 1) normalize raw reads among samples, 2) separate transcriptome profiles within and between KO models and 3) identify differentially expressed APSs (DE-APSs) with Padj (corrected) < 0.1. All APSs, including the DE-APSs were further annotated using the HOMER (v4.9) software suite (http://biowhat.ucsd.edu/homer/) to determine mapping locations, including TTS (transcription terminal site, by default defined from −100 bp to +1 kb), exons, introns, 3′UTR, 5′UTR, TSS (transcription start site, by default defined from −1kb to +100 bp) and intergenic regions^35^. The protein coding genes associated with the DE-APSs were then used as input in the Metascape program and pathways were enriched using “GO Biological Processes”^8^. Protein-protein interaction networks were identified using the STRING database (https://string-db.org/)^36^ and visualized using the graphical network Cytoscape software (version 3.5.1.)^37^. χ^2^ test was used to detect significant differences among or between categories using R. In addition, the raw WTTS-seq and WTSS-seq  reads were deposited in NCBI GEO (gene expression omnibus) database under accession numbers GSE107212 and GSE112325.

## Electronic supplementary material

Dataset 1

Dataset 2A

Dataset 2B

Dataset 2C

Dataset 2D

Dataset 2E

Dataset 2F

Dataset 2G
