# Supplementary material for: Alternative polyadenylation drives genome-to-phenome information detours in the AMPKα1 and AMPKα2 knockout mice
Source: Sci Rep. 2018 Apr 24;8:6462. doi: 10.1038/s41598-018-24683-7 (PMC5915415; doi:10.1038/s41598-018-24683-7)
Supplement: Supplementary file 1 — Dataset 1 [file 41598_2018_24683_MOESM1_ESM.docx]

**Alternative polyadenylation drives genome-to-phenome information detours in the AMPKα1 and AMPKα2 knockout mice**

Shuwen Zhang, Yangzi Zhang, Xiang Zhou, Xing Fu, Jennifer J. Michal, Guoli Ji, Min Du, Jon F. Davis and Zhihua Jiang


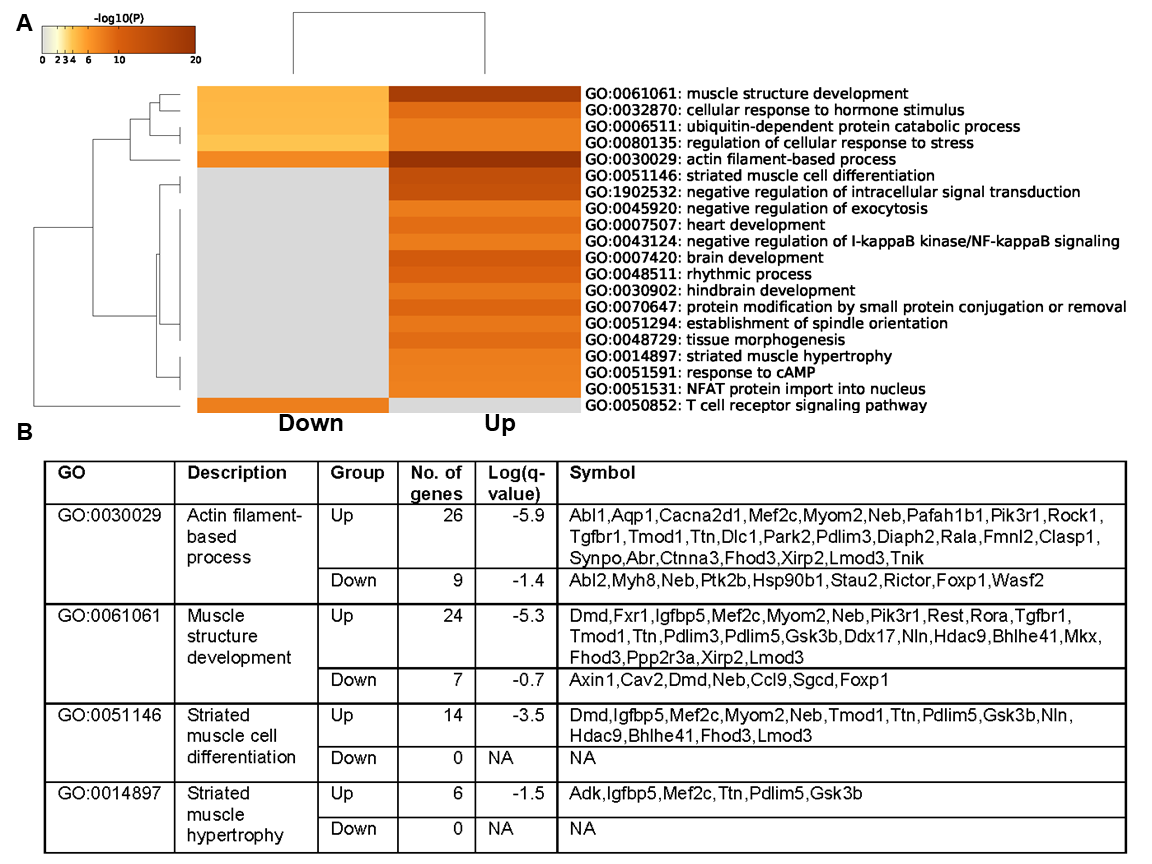


**Supplementary Figure S1. Characterization of alternative transcriptional start sites in AMPKα2 knockout mice.**  (A) Pathway enrichment with differentially expressed alternative start sites up- and down-regulated in AMPKα2 knockout mice. (B) Lists of De-genes contributed to the muscle related pathways.


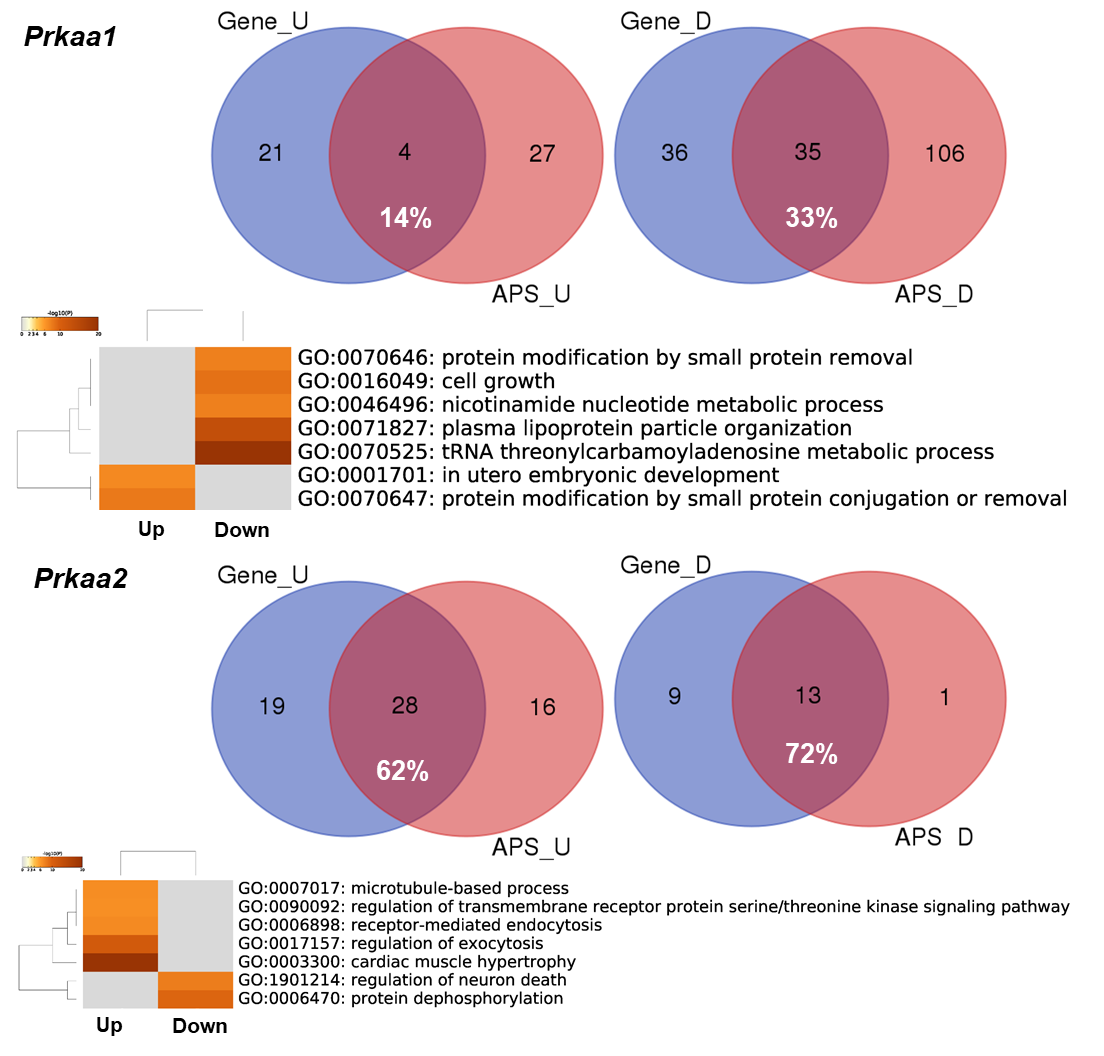


**Supplementary Figure S2. Gene-based analysis and pathway enrichment.**  Differences in numbers of DE-genes between gene- and APS-based analysis and gene-based pathways in AMPKα1 knockout mice (A) and in AMPKα2 knockout mice (B). U: up-regulated; and D: down-regulated.
