# Supplementary material for: Alternative polyadenylation drives genome-to-phenome information detours in the AMPKα1 and AMPKα2 knockout mice
Source: Sci Rep. 2018 Apr 24;8:6462. doi: 10.1038/s41598-018-24683-7 (PMC5915415; doi:10.1038/s41598-018-24683-7)
Supplement: Supplementary file 8 — Dataset 2G [file 41598_2018_24683_MOESM8_ESM.docx]

**Alternative polyadenylation drives genome-to-phenome information detours in the AMPKα1 and AMPKα2 knockout mice**

Shuwen Zhang, Yangzi Zhang, Xiang Zhou, Xing Fu, Jennifer J. Michal, Guoli Ji, Min Du, Jon F. Davis and Zhihua Jiang

**Table S7. Characterization of up- and down-regulated DE-APSs between KO and WT mice using either Cuffcompare or HOMER tool.**

|  | | Conventional APS  (c, o and p) | Non-conventional APSs (e, i and x) |
| --- | --- | --- | --- |
| Whole Dataset | | **47%** | **53%** |
| AMPKα1 KO mice | up | 42%↓ | 58%↑ |
|  | down | 82%↑ | 18%↓ |
| AMPKα2 KO mice | up | 18%↓ | 82%↑ |
|  | down | 93%↑ | 7%↓ |

|  | | Conventional APS  (3’UTR and TTS) | Non-conventional APSs (exon, intron etc.) |
| --- | --- | --- | --- |
| Whole Dataset | | **47%** | **53%** |
| AMPKα1 KO mice | up | 32%↓ | 68%↑ |
|  | down | 75%↑ | 25%↓ |
| AMPKα2 KO mice | up | 6%↓ | 94%↑ |
|  | down | 93%↑ | 7%↓ |
